# Hybridization chain reaction-assisted CRISPR/Cas12a strategy for rapid and visual detection of *Haemophilus influenzae*

**DOI:** 10.3389/fcimb.2026.1844708

**Published:** 2026-07-07

**Authors:** Chunfang Ma, Jiayi Zhang, Yayun Jiang, Xiangxiang Li

**Affiliations:** 1 Department of Clinical Laboratory, Suzhou Ninth People’s Hospital, Suzhou Ninth Hospital Affiliated to Soochow University, Suzhou, China; 2 Department of Obstetrics, Suzhou Ninth People’s Hospital, Suzhou Ninth Hospital Affiliated to Soochow University, Suzhou, China; 3 Department of Clinical Laboratory, Deyang People′s Hospital, Deyang, China

**Keywords:** CRISPR/Cas12a, *Haemophilus influenzae*, hybridization chain reaction, rapid, visual detection

## Abstract

*Haemophilus influenzae* (*H. influenzae*) is a major pathogen causing community-acquired pneumonia in children, posing a serious threat to children’s health. Rapid and convenient testing is needed for effective treatment. Traditional detection methods, such as bacterial culture and qPCR are cumbersome to operate, and require sophisticated instrumentation. Here, we developed a visual CRISPR/Cas detection platform that integrates the hybridization chain reaction (HCR) and horseradish peroxidase (HRP)-catalyzed 3,3′,5,5′-Tetramethylbenzidine (TMB) colorimetric change, called Vi-CasHCP. The single-base recognition capability of CRISPR/Cas12a improves the specificity, while the efficiency of HCR shortens the detection time and improves the sensitivity. The peroxidase-like activity of HRP catalyzes the oxidation of TMB to oxTMB, resulting in a blue color change. Vi-CasHCP achieved a detection limit of 11.8 CFU/mL in 70 min, after Recombinase polymerase amplification. Clinical validation using 50 respiratory samples showed complete diagnostic agreement with qPCR, with 100% sensitivity, specificity, PPV, and NPV. Therefore, this platform offers rapid detection, requires no complex instruments, and has high sensitivity, providing fast and accurate diagnostic support for clinical practice, and is particularly suitable for resource-limited settings.

## Introduction

*Haemophilus influenzae* (*H. influenzae*) is a major pathogen in community-acquired pneumonia in children (Ulanova and Tsang, 2014; Zhou et al., 2023). It can breach the mucosal barrier and enter the bloodstream, leading to invasive infections such as sepsis, purulent meningitis, and purulent osteoarthritis, posing a serious threat to children’s life and health (Gao et al., 2023; De Angelis et al., 2025). Accurate and timely early diagnosis helps identify infected children, guide precise anti-infective treatment, halt disease progression, and prevent serious complications, thereby effectively safeguarding children’s health (Yao et al., 2026). However, conventional *H. influenzae* detection methods, such as bacterial culture and polymerase chain reaction (PCR), are labor-intensive, time-consuming, and reliant on expensive instrumentation (Diallo et al., 2021; Marasini et al., 2022). This limits their use in early clinical screening and primary healthcare (Short et al., 2021). Therefore, there is an urgent need to develop a new detection technology that is fast, simple, and efficient.

Clustered regularly interspaced short palindromic repeats (CRISPR) and CRISPR-associated (Cas) systems are acquired immune systems that bacteria and archaea have evolved to resist invasion by exogenous substances such as bacteriophages and plasmids (Marraffini, 2015; Phan et al., 2022). The CRISPR/Cas can precisely identify and cleave specific DNA or RNA sequences, and has become an important tool for transcriptional regulation, gene editing, and DNA/RNA detection (Ji et al., 2025; Wei et al., 2025). Among them, Cas12a, guided by crRNA, binds to target dsDNA to form a ternary complex, specifically recognizing and cleaving the target dsDNA, while simultaneously performing non-specific cleavage of ssDNA within the system (Mao et al., 2022; Che et al., 2025). Cas12a features a mild response, no requirement for thermal cycling, and strong signal amplification, making it a promising candidate for nucleic acid biosensor development (Li et al., 2021; Jiang et al., 2026). Based on this characteristic, Doudna’s team developed the DETECTR system by using the FAM-ssDNA-BHQ1 fluorescent probe to output signals, enabling genotyping of high-risk human papillomaviruses HPV18 and HPV16 (Chen et al., 2018). Jin Wang’s team developed the HOLMES detection system using Cas12a and HEX-ssDNA-BHQ1 fluorescent probes, demonstrating reliable, rapid detection of pseudorabies virus and Japanese encephalitis virus (Li et al., 2018). These detection systems clearly demonstrate the broad potential of CRISPR/Cas12a for biosensing.

In our previous research, we developed a rapid and highly sensitive fluorescence detection method for Methicillin-resistant *Staphylococcus aureus* (MRSA), termed MCFHCR, by integrating CRISPR/Cas12a with the signal-amplification effect of the Hybridization Chain Reaction (HCR) (Jiang et al., 2025). HCR is a powerful signal amplification technique based on DNA strand displacement, operating without enzyme catalysis and under isothermal conditions (Zhang et al., 2021; Jiang et al., 2023). The HCR system consists of complementary DNA hairpins (H1, H2) with sticky ends and a trigger (H0) (Dirks and Pierce, 2004; Duan et al., 2020). Through a series of hybridization reactions, it generates functional nucleic acids containing alternating copolymers, enabling highly sensitive detection in a short time (Zheng et al., 2025).

The MCFHCR platform demonstrates high sensitivity, achieving a limit of detection (LOD) of 5 copies/μL for *mecA* DNA. However, the signal output of these methods depends mainly on probe fluorescence, which is susceptible to photobleaching and background noise. Moreover, fluorescence-based detection requires specialized light sources and instrumentation, which limits its broader applicability.

Visual detection does not require complex instruments, as the presence of a target can be determined simply by observing signal changes. Among visual detection strategies, lateral flow assays (LFAs) are the most widely employed for rapid on-site applications, owing to their simplicity and portability. However, their relatively low sensitivity, limited quantitative capability, and suboptimal specificity restrict their broader applicability. Colorimetric visual detection enables the direct identification of analytes through observable color changes, offering advantages such as rapid response, operational simplicity, real-time monitoring, and low cost (Broughton et al., 2020; Liu et al., 2025). Integrating the CRISPR/Cas system with colorimetric detection eliminates the need for sophisticated instrumentation (Ki et al., 2022). It reduces the technical expertise required of operators, thereby broadening the range of applications and enhancing their potential (Ding et al., 2020). 3,3’,5,5’-Tetramethylbenzidine (TMB) is the most commonly used chromogenic substrate (Li et al., 2025). Its colorimetric reaction relies on peroxidase-mediated oxidation of colorless TMB into colored products in the presence of H_2_O_2_, producing a visible color change.

Herein, based on our previous work, we developed a convenient and rapid visual nucleic acid colorimetric detection platform, termed Vi-CasHCP, for detecting *H. influenzae*. By utilizing the specific cleavage capability of CRISPR/Cas12a, the signal amplification of HCR, and the peroxidase-like activity of HRP, the sensitivity of *H. influenzae* detection were significantly improved. HRP catalyzed the oxidation of TMB to oxTMB, producing a color change that enabled simple and rapid colorimetric detection. Vi-CasHCP provides a novel solution for the early diagnosis of *H. influenzae*, offering significant clinical value and a new strategy for rapid resource-limited settings. Furthermore, given the editability of CRISPR/Cas12a, Vi-CasHCP can be extended to detect other respiratory pathogens, demonstrating strong potential for application (Rahimi et al., 2021).

## 2 Experimental section

### 2.1 Materials and reagents

TMB liquid substrate system (containing 3,3′,5,5′-tetramethylbenzidine and peroxide solution), Stop Solution for TMB Substrate (650nm), bovine serum albumin (BSA), and TBE PAGE Gel Preparation Kit were provided from Beyotime Biotechnology Co., Ltd. (Shanghai, China)., Ltd. LbCas12a and 10 × isothermal amplification Buffer were acquired from New England Biolabs Inc. (United States). Carboxyl-coated magnetic nanoparticles were purchased from PuriMag Biotechnology Co., Ltd. (Xiamen, China). DNase/RNase-free H_2_O, Tris-EDTA Buffer (TE), and Diethylpyrocarbonate (DEPC) water were purchased from Beijing Solarbio Biotechnology Co., Ltd. (Beijing, China). All oligonucleotides (HPLC-purified) and recombinant plasmids were synthesized by Sangon Biotech Co., Ltd. (Shanghai, China) (**Supplementary Table 1**). The RPA Kit, a recombinase polymerase amplification nucleic acid amplification kit, was obtained from GenDx Biotech Co., Ltd. (Suzhou, China). *Haemophilus influenzae* (*H. influenzae*, ATCC9007 and ATCC49247), *Pseudomonas aeruginosa* (*P. aeruginosa*, ATCC27853), *Streptococcus pneumoniae* (*S. pneumoniae*, ATCC49619), *Escherichia coli* (*E. coli*, ATCC 25922), *Streptococcus pyogenes* (*S. pyogenes*, ATCC 19615), *Klebsiella pneumoniae* (*K. pneumoniae*, ATCC700603), and *Staphylococcus aureus* (*S. aureus*, ATCC29213), *Moraxella catarrhalis* (*M. catarrhalis*, ATCC 25240) were laboratory-preserved strains.

### 2.2 Preparation of MNP-H0

Add 200 μL of coupling buffer (50 mM MES, pH 6.0, 0.01% Triton X-100) to 40 μL of 10 mg/mL carboxyl-modified MNPs, and wash the magnetic beads three times using a magnetic rack. Next, add 80 μL of coupling buffer to resuspend the MNPs, followed by 8 μL of 100 μM NH_2_-H0. Shake and mix the solution at room temperature for 30 minutes. Then add 40 μL of 50 mg/mL EDC•HCl solution to the reaction mixture and incubate at room temperature with shaking for 4 hours. Finally, perform magnetic separation and wash the mixture three times to obtain MNP-H0.

### 2.3 Verification of the CRISPR/Cas12a system

Three crRNA sequences targeting the *H. influenzae*-specific gene outer membrane protein P6 gene (*omp6*) (Cao et al., 2021; Wang et al., 2022), along with an FQ-labeled probe, were used to verify the feasibility of the CRISPR/Cas12a detection system. The reaction mixture was prepared by combining 4 µL of 1 µM Cas12a, 20 µL of 200 nM crRNA, 10 µL of 1 µM FQ probe, 2 µL of PCR amplification product, and 4 µL of 10 × NEBuffer. The mixture was incubated at 37 °C for 30 minutes. Fluorescence intensity changes in the FAM channel were monitored using the Hongshi SLAM-96S Real-Time PCR instrument. In the bead-based DNA cleavage assay, the FQ probe was replaced with MNP-H0, while the amounts of the other components remained the same.

### 2.4 HCR-mediated signal amplification

Based on the Complementary Nucleobase Interactions and the sequence of the initiator strand H0, the hairpin structures H1 and H2 required for the HCR reaction were designed. The secondary structures of H1, H2, and the reaction products were analyzed online with Unfold, and the changes in Gibbs free energy before and after the reaction were calculated. H1 and H2 were dissolved to the desired concentration, heated at 95 °C for 5 minutes, and then allowed to cool naturally to room temperature to facilitate the formation of stable hairpin structures. Add 0.1 μM H0 to a mixture of H1 and H2 (each at 1 μM), and adjust the volume to 20 μL with HCR buffer. The reaction mixture was incubated at 37 °C for 20 minutes. HCR-mediated signal amplification was verified using 12% polyacrylamide gel electrophoresis (PAGE).

### 2.5 Detection of target DNA by Vi-CasHCP

Add 2 μL of the MNP-H0 probe to the CRISPR/Cas12a reaction mixture and incubate at 37 °C in a metal bath for 30 minutes. Following this, perform HCR amplification directly on the magnetic beads. After completion of the reaction, apply magnetic separation to isolate the beads. Fresh HRP-conjugated reagents were prepared and pre-validated for catalytic activity prior to each experiment. Next, add the HRP solution to the beads and incubate at room temperature for 3 minutes, followed by three wash steps to remove unbound reagents. Subsequently, add 100 μL of TMB substrate solution to the magnetic beads, vortex thoroughly, briefly centrifuge, and incubate at room temperature in the dark for 3 minutes. Terminate the reaction by adding 50 μL of stop solution. Finally, measure the absorbance of the solution across the wavelength range of 340–700 nm using a microplate reader. The colorimetric results were interpreted according to the reader guideline provided in the **Supplementary Materials**. Each experiment included both negative and positive controls to monitor baseline signals and ensure that the observed changes were specific to target-mediated reactions.

### 2.6 Bacterial culture and genomic DNA extraction

Respiratory tract specimens were inoculated onto blood agar plates and chocolate agar plates using sterile disposable inoculation loops. The plates were incubated at 37 °C for 18–24 hours in a 5% CO_2_ incubator. Colonies suspected to be *H. influenzae* were selected and subcultured onto chocolate agar plates, while other strains were subcultured onto blood agar plates. All plates were further incubated under the same conditions for 18–24 hours to isolate and purify the strains. All clinical strains were identified using microbial mass spectrometry. Bacterial genomic DNA was extracted using the TaKaRa MiniBEST Bacterial Genomic DNA Extraction Kit for the performance evaluation of the Vi-CasHCP assay. For clinical strain detection, genomic DNA was extracted using the boiling lysis method: samples were incubated in a boiling water bath for 10 minutes, and the supernatant was collected for direct use in the assay.

### 2.7 Evaluation of analytical performance

The standard strain ATCC 49247 was used as a representative of *H. influenzae* to evaluate the sensitivity, selectivity, and other performance characteristics of the Vi-CasHCP visualization detection method. The bacterial suspension was adjusted to an optical density (OD) of 0.5, as per the McFarland standard, using a turbidimeter. The suspensions were serially diluted with PBS, inoculated onto agar plates, and colony counts performed to determine the bacterial concentration. The ATCC 49247 strain was serially diluted to concentrations ranging from 6 to 6 × 10^4^ CFU/mL and detected using the Vi-CasHCP visual assay to evaluate its analytical performance. To assess the specificity of the Vi-CasHCP method, ATCC 49247 at 10^4^ CFU/mL and common respiratory tract bacterial strains were tested. All experiments were performed in triplicate.

### 2.8 Clinical strain and clinical sample testing

Clinical strains and specimens were obtained from Deyang People’s Hospital and Suzhou Ninth People's Hospital, including 50 respiratory tract samples and 20 clinically isolated strains. All clinical strains and specimens were collected with approval from the Medical Research Ethics Committee of Suzhou Ninth People's Hospital and Deyang People’s Hospital. The ethical review number is KYLW2026-029–01 and 2025-04-002-K01. Clinical information, including sample type, patient age, clinical diagnosis, and clinical test results, is provided in **Supplementary Table 3** and **S4**. Each sample underwent routine clinical testing, including bacterial culture and mass spectrometry identification, and a clinical test report was issued. This procedure ensured that all samples used to evaluate the Vi-CasHCP platform were accurately characterized and suitable for comparison with the qPCR reference method.

To evaluate the Vi-CasHCP platform’s detection performance in clinical applications, all respiratory samples were tested, and the results were validated using real-time quantitative PCR (qPCR). The qPCR reaction mixture, with a total volume of 30 μL, consisted of 5 μL of DNA template and 25 μL of PCR master mix. The amplification protocol was as follows: initial denaturation at 94 °C for 5 minutes, followed by 40 cycles of denaturation at 94 °C for 15 seconds and annealing at 58 °C for 1 minute. Fluorescence signals in the FAM channel were collected during the annealing step of each cycle. The fluorescence threshold was automatically set by the real-time PCR software as 10 times the standard deviation of the baseline fluorescence signal. The Ct values were calculated using real-time PCR software to assess the detection results.

## 3 Results and discussion

### 3.1 Principle of the Vi-CasHCP platform

**Figure 1:**
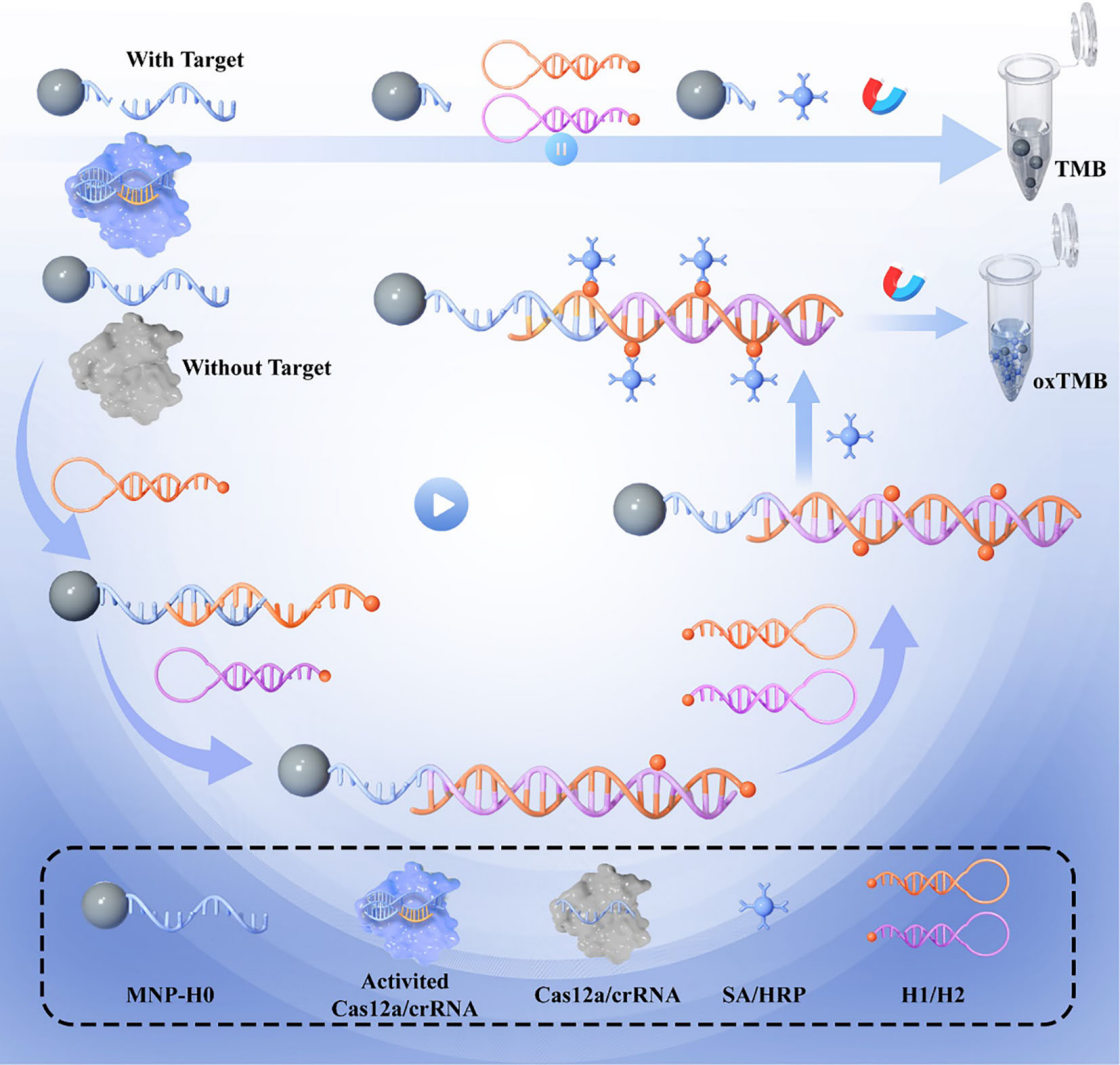
Schematic illustration of the Vi-CasHCP platform.

The Vi-CasHCP colorimetric visualization detection scheme consists of three parts: target gene activation of CRISPR/Cas12a trans-cleavage activity, DNA functionalized magnetic bead-mediated HCR reaction, and HRP-catalyzed TMB-H_2_O_2_ reaction. The DNA-functionalized magnetic beads, which link target recognition and signal output, are the core component of Vi-CasHCP. As shown in **Figure 1**, when *omp6* is present, CRISPR/Cas12a is activated, and the H0 is nonspecifically cleaved. As a result, the biotin-modified hairpins H1 and H2 cannot open, preventing the HCR reaction from proceeding. Magnetic beads cannot capture avidin-modified HRP (SA-HRP). After magnetic separation, SA-HRP remains free in the solution, and the magnetic beads cannot catalyze the TMB-H_2_O_2_ reaction, so the solution does not change color. In contrast, when the target gene is absent, CRISPR/Cas12a is inactivated, and the H0 coupled to the magnetic bead surface remains intact. H0, as a substrate chain, hybridizes with biotin-modified hairpin H1, exposing new sticky ends to open biotin-modified hairpin H2. After a series of hybridizations, a large number of biotin-modified DNA double strands are formed and incubated with SA-HRP, which couples a large number of SA-HRPs to the surface of the magnetic beads. This catalyzes the TMB-H_2_O_2_ reaction, turning the solution from colorless to blue, ultimately enabling visual colorimetric detection.

### 3.2 Validation of the Vi-CasHCP platform

**Figure 2:**
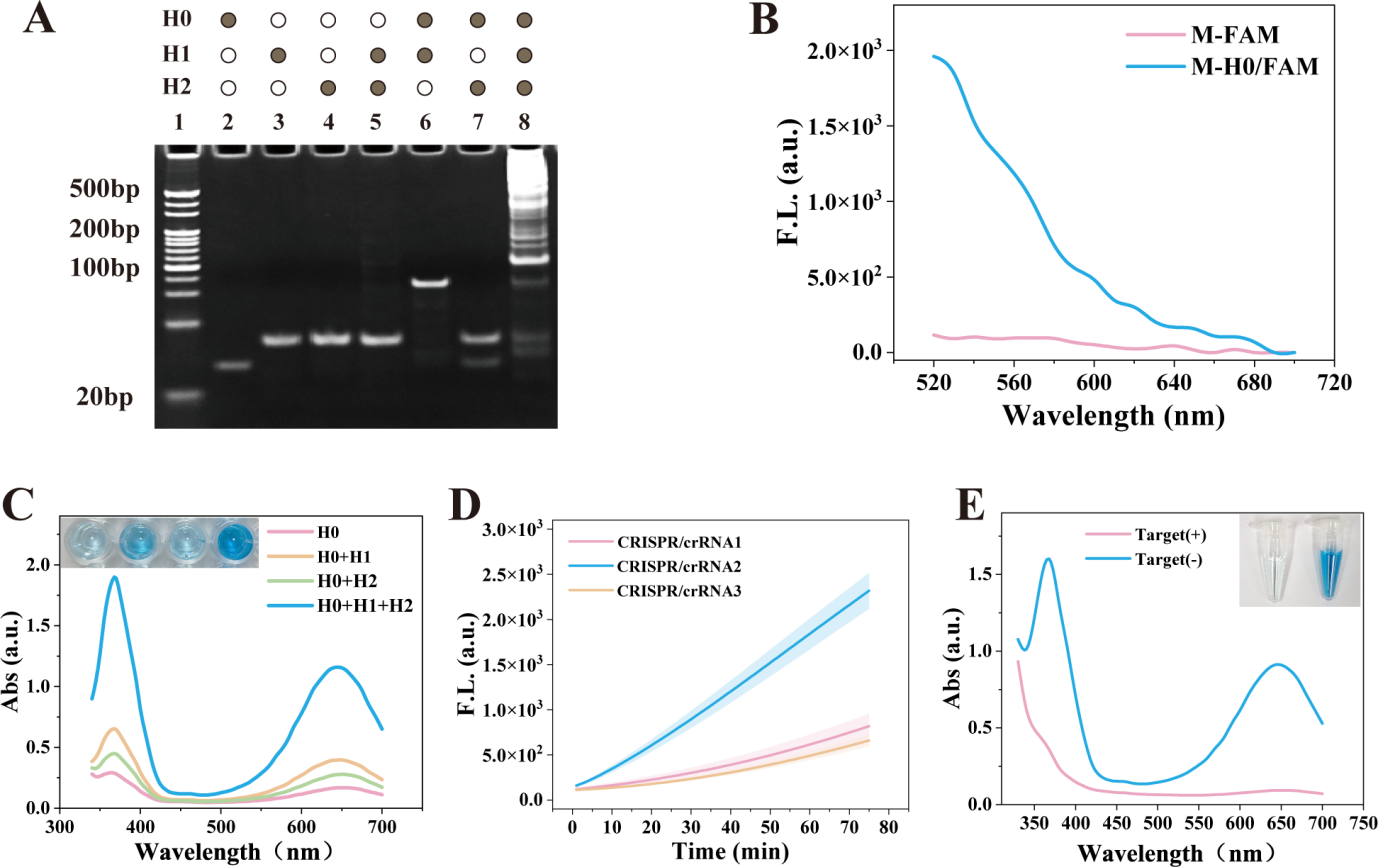
Validation of the Vi-CasHCP Platform. **(A)** Verification of HCR feasibility by 12% polyacrylamide gel electrophoresis. Lanes 1-8: Marker, H0, H1, H2, H1+H2, H0+H1, H0+H2, H0+H1+H2, respectively. **(B)** FAM-modified DNA hybridization demonstrates the successful preparation of DNA-functionalized magnetic beads. **(C)** UV-Vis absorption spectroscopy proves the HCR on the surface of magnetic beads. **(D)** Dynamic fluorescence changes of ssDNA-Probe cleaved by CRISPR/Cas12a. **(E)** The feasibility of detecting *H. influenzae* using the Vi-CasHCP platform by UV-Vis absorption spectrum.

We sequentially verified each key step of the Vi-CasHCP platform. First, a H0 with 12 T bases added to its 5’ end was designed to prevent steric hindrance during cleavage on the magnetic bead surface. Based on the principle of nucleic acid base pairing, we designed two metastable DNA hairpin structures, H1 and H2 (**Supplementary Table 2**). To avoid nonspecific amplification, we used NUPACK software to simulate DNA secondary structure at 37 °C and predict Gibbs free energy online. Subsequently, the feasibility of the HCR reaction in solution was verified by polyacrylamide gel electrophoresis. As shown in **Figure 2A**, when H0 and H1 coexisted (Lane 6), a new band appeared, indicating that H0 could hybridize with H1. Upon the simultaneous introduction of H0, H1, and H2 (Lane 7), a characteristic ladder-like pattern appeared, confirming successful HCR initiation and the formation of long DNA polymers. To verify the successful preparation of MNPs-H0, we designed a FAM-modified single-stranded DNA complementary to H0 and hybridized it with the DNA-functionalized magnetic beads. As shown in **Figure 2B**, the fluorescence intensity of the DNA-functionalized bead group (M-H0) was significantly higher than that of the control group (M). To further confirm DNA conjugation, we measured the zeta potential of the magnetic beads before (M) and after (M-H0) modification. As illustrated in **Supplementary Figure 1**, the zeta potential decreased following DNA conjugation, consistent with the negatively charged phosphate backbone of DNA. Together, the fluorescence and zeta potential measurements provide strong evidence for the successful surface functionalization of the magnetic beads. Then, we demonstrated that MNP-H0 can initiate the HCR reaction by UV-Vis absorption spectroscopy. The results are shown in **Figure 2C**, where the absorbance is strongest at 650 nm when both H1 and H2 are present. The cleavage efficiency of Cas12a directly affects the detection performance of the Vi-CasHCP platform. Three crRNAs targeting the *H. influenzae*-specific *omp6* gene were designed (**Supplementary Figure 1**), and the most efficient crRNA2 was selected using fluorescence probe-based assays (**Figure 2D**). Finally, the feasibility of colorimetric detection on the Vi-CasHCP platform was validated. No significant color change was observed in the Target (+) group, whereas the Target (-) group turned visibly blue, demonstrating the feasibility of detection with the Vi-CasHCP platform (**Figure 2E**).

### 3.3 Optimization of experimental conditions for the Vi-CasHCP platform

**Figure 3:**
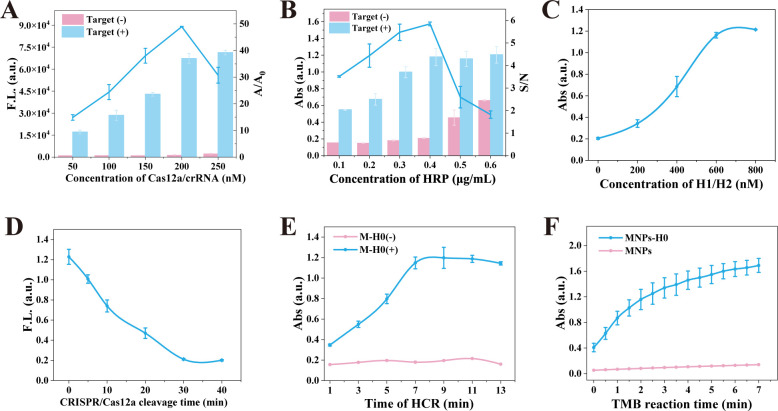
Optimization of experimental conditions for the Vi-CasHCP platform. Three biological replicates (n = 3) were performed per concentration. **(A)** Optimization of Cas12a/crRNA concentration. Five concentrations (50, 100, 150, 200, 250 nM) were tested in Target(-) and Target(+) groups. A0: Fluorescence intensity of group without target, A: Fluorescence intensity of group with target **(B)** Optimization of HRP concentration. Six concentrations (0.1, 0.2, 0.3, 0.4, 0.5, 0.6 μg/mL) were tested in Target(-) and Target(+) groups, with three biological replicates (n = 3) per concentration. N: Absorbance value of group without target, S: Absorbance value of group with target. **(C)** Optimization of H1/H2 concentration for HCR amplification. Six concentrations (0, 100, 200, 400, 600, 800 nM) were tested, with three biological replicates (n = 3) per concentration. **(D)** Optimization of CRISPR/Cas12a cleavage time. Reaction times of 5, 10, 20, 30, and 40 min were evaluated, with three replicates (n = 3). **(E)** Investigation of the optimal time for HCR. Positive (M-H0(+), containing initiator H0) and negative (M-H0(-), without H0) groups were tested for 1, 3, 5, 7, 9, 11, and 13 min, with three replicates (n = 3). **(F)** Optimization of TMB color development time. Positive (MNPs-H0) and negative (MNPs) groups were evaluated for 0–7 min, with three replicates (n = 3).

To enhance the performance of the Vi-CasHCP platform, we systematically optimized the concentrations of Cas12a/crRNA, H1/H2, and HRP, along with the Cas12a cleavage time, HCR reaction time, and TMB color development time. The concentration of Cas12a/crRNA has a significant impact on the cleavage efficiency of CRISPR/Cas12a. We first established Target(-) and Target(+) groups to evaluate the cleavage efficiency of Cas12a/crRNA (**Figure 3A**). Reactions were performed at five Cas12a/crRNA concentrations (50, 100, 150, 200, and 250 nM), with three biological replicates (n=3) per concentration. Results indicated that 200 nM produced the highest fluorescence signal-to-noise ratio and was thus selected for subsequent experiments. As a catalyst, HRP concentration directly affects the colorimetric reaction, and excessively high concentrations may increase the background signal. Using the same grouping, HRP concentrations were optimized at six levels: 0.1, 0.2, 0.3, 0.4, 0.5, and 0.6 μg/mL, with three biological replicates (n=3) per level. Results demonstrated that 0.4 μg/mL HRP achieved the highest signal-to-noise ratio on the Vi-CasHCP platform (**Figure 3B**). Higher HCR amplification efficiency results in more HRP binding sites. To enhance colorimetric performance, we optimized the concentrations of H1 and H2. As shown in **Figure 3C**. As H1/H2 concentrations increased from 0 to 600 nM, absorbance gradually rose. Beyond 600 nM, the increase slowed, indicating that higher concentrations had limited additional effect on signal enhancement. To reduce detection time while maintaining performance, Cas12a cleavage time, HCR reaction time, and TMB color development time were optimized. Cas12a cleavage was evaluated for up to 40 min (**Figure 3D**). Results showed that fluorescence reached a maximum and stabilized at a cleavage time of 30 min. HCR reaction time was optimized using a positive group (M-H0(+), containing initiator chain H0) and a negative group (M-H0(-), without the initiator chain). Absorbance of the positive group increased with HCR reaction time and stabilized at 7 min, while the negative group remained low throughout. This indicates that 7 min is the optimal HCR reaction time, balancing detection efficiency and signal intensity (**Figure 3E**). Finally, TMB color development was assessed for 0–7 min. The MNPs-H0 group plateaued at 3 min, indicating that 3 min is optimal for sufficient signal while preventing background accumulation from excessively long reactions (**Figure 3F**). Therefore, we selected these optimal experimental conditions for further investigation of detection performance.

### 3.4 Analytical performance of the Vi-CasHCP platform

**Figure 4:**
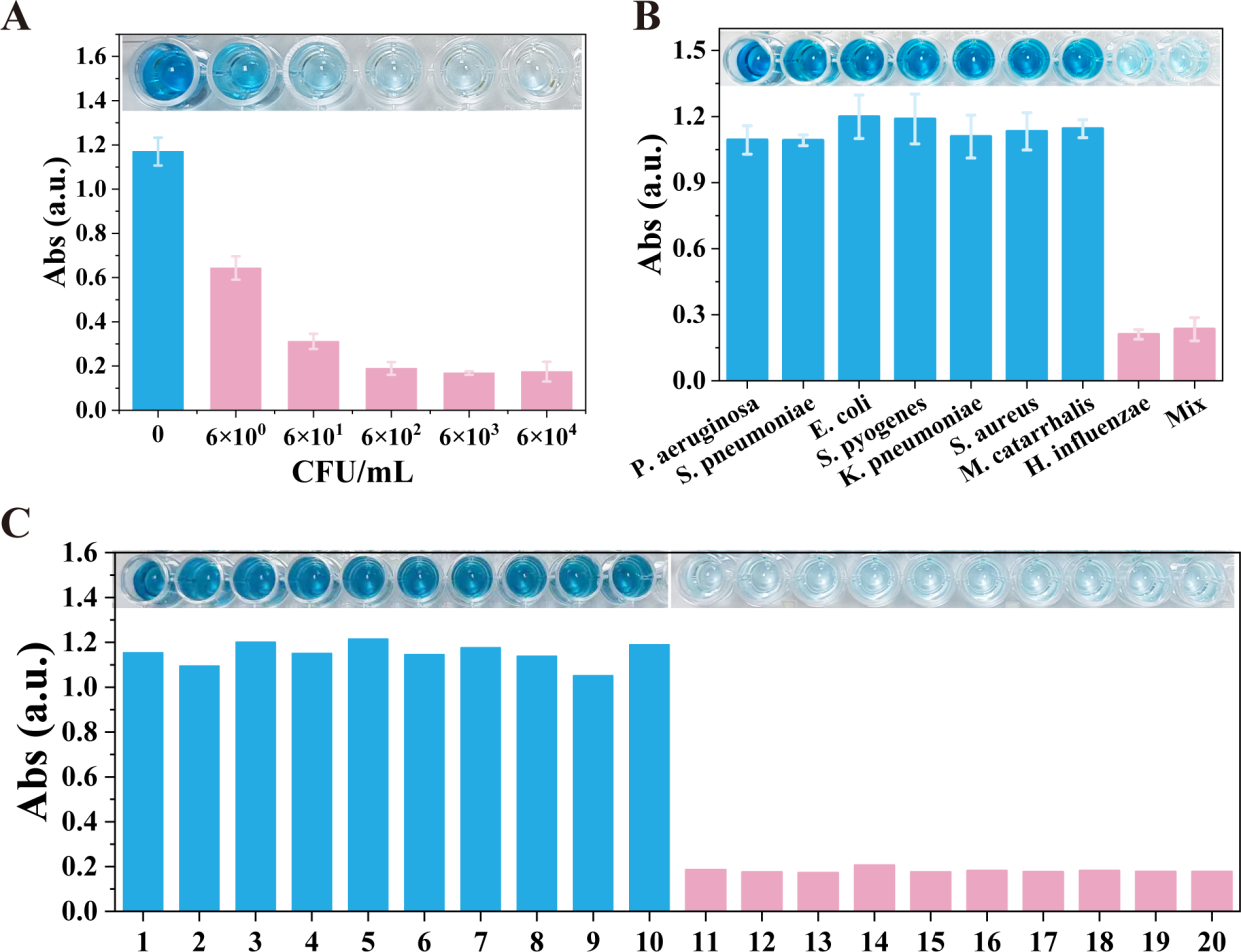
Analytical performance of the Vi-CasHCP platform. **(A)** Sensitivity analysis (Top: the photograph of detection results; Bottom: Absorbance at 650 nm of Vi-CasHCP response to different concentrations of *H. influenzae*). **(B)** Specificity analysis (Top: the photograph of detection results; Bottom: Absorbance at 650 nm of Vi-CasHCP response to different standard strains). **(C)** Clinical strain detection, strain 1-10: *P. aeruginosa*, *S. pneumoniae*, *K. pneumoniae*, *M. catarrhalis*, *M. catarrhalis*, *E. coli*, *S. aureus*, *E. coli*, *S. pneumoniae*, *K. pneumoniae*. 11-20: *H. influenzae* (Top: the photograph of detection results; Bottom: Absorbance at 650 nm of Vi-CasHCP response to different clinical respiratory tract isolates).

To evaluate the detection performance of the Vi-CasHCP platform, we systematically assessed its sensitivity and specificity. First, four pairs of RPA primers targeting the *omp6* gene sequence were designed and synthesized. The primer combination with the best amplification efficiency was selected using 3% agarose gel electrophoresis (**Supplementary Figure 3**). To determine the threshold for Vi-CasHCP, we tested multiple non-target genes to assess the background signal for colorimetric detection (**Supplementary Figure 4**). Vi-CasHCP is a signal-off detection platform: negative samples remain blue with high absorbance, whereas positive samples, due to disruption of the initiation chain H0, fail to trigger the HCR reaction, thereby inhibiting the colorimetric response. Accordingly, the threshold for positive detection was set as the mean of negative control samples (X) minus three standard deviations (SD), which corresponded to 1.03. Subsequently, we validated the performance detection of Vi-CasHCP using *H. influenzae* genomic DNA at concentrations ranging from 6 to 6 × 10^4^ CFU/mL (**Figure 4A**). The results showed that absorbance at 650 nm decreased progressively with increasing bacterial concentration, accompanied by pronounced colorimetric changes. Furthermore, analytical sensitivity testing was systematically conducted using four sets of diluted samples, with concentrations of 12, 9, 6, and 3 CFU/mL. Each concentration was tested in 20 replicates (**Supplementary Table 5**). The test results showed that for *H. influenzae*, all 20 replicates were positive at 12 CFU/mL; 17 out of 20 replicates were positive at 9 CFU/mL; and 13 and 4 out of 20 replicates were positive at 6 and 3 CFU/mL, respectively (**Supplementary Table 6**). The limit of detection (LOD) at 95% confidence was determined by weighted Probit regression. The estimated LOD was 11.8 CFU/mL (95% CI: 9.8 – 14.3 CFU/mL).

To evaluate the specificity of the Vi-CasHCP platform, we selected several standard strains responsible for common respiratory infections, including *P. aeruginosa* (ATCC27853), *S. pneumoniae* (ATCC49619), *E. coli* (ATCC 25922), *S. pyogenes* (ATCC 19615), *K. pneumoniae* (ATCC700603), and *S. aureus* (ATCC29213). M. catarrhalis (ATCC 25240). We first verified the *omp6* gene amplification in these strains by 3% agarose gel electrophoresis, demonstrating that *omp6* amplification was observed only in *H. influenzae* (**Supplementary Figure 5**). The Vi-CasHCP assay also yielded consistent results. As shown in **Figure 4B**, all non-target strains produced a distinct blue color. In contrast, samples containing the *H. influenzae omp6* target gene showed no significant color change. In addition, we collected 20 clinical isolates to verify the detection performance of Vi-CasHCP (**Supplementary Table 3**). We verified the *P6* gene amplification in *H. influenzae* (10 strains), *S. pneumoniae* (2 strains), *S. aureus* (1 strain), *E. coli* (2 strains), *P. aeruginosa* (1 strain), *K. pneumoniae* (2 strains), and *M. catarrhalis* (2 strains) using 3% agarose gel electrophoresis (**Supplementary Figure 6**). Vi-CasHCP test results showed that the control strains turned distinctly blue, while *H. influenzae* did not exhibit any obvious color change, (**Figure 4C**). These results demonstrate that the Vi-CasHCP platform exhibits high sensitivity and is capable of specifically distinguishing *H. influenzae* from other common respiratory pathogens. Finally, to assess the batch-to-batch reproducibility of M-H0, four independently prepared batches were tested under the same conditions. The absorbance at 650 nm demonstrated good preparation consistency and reproducibility among different batches (**Supplementary Figure 7**).

**Figure 5:**
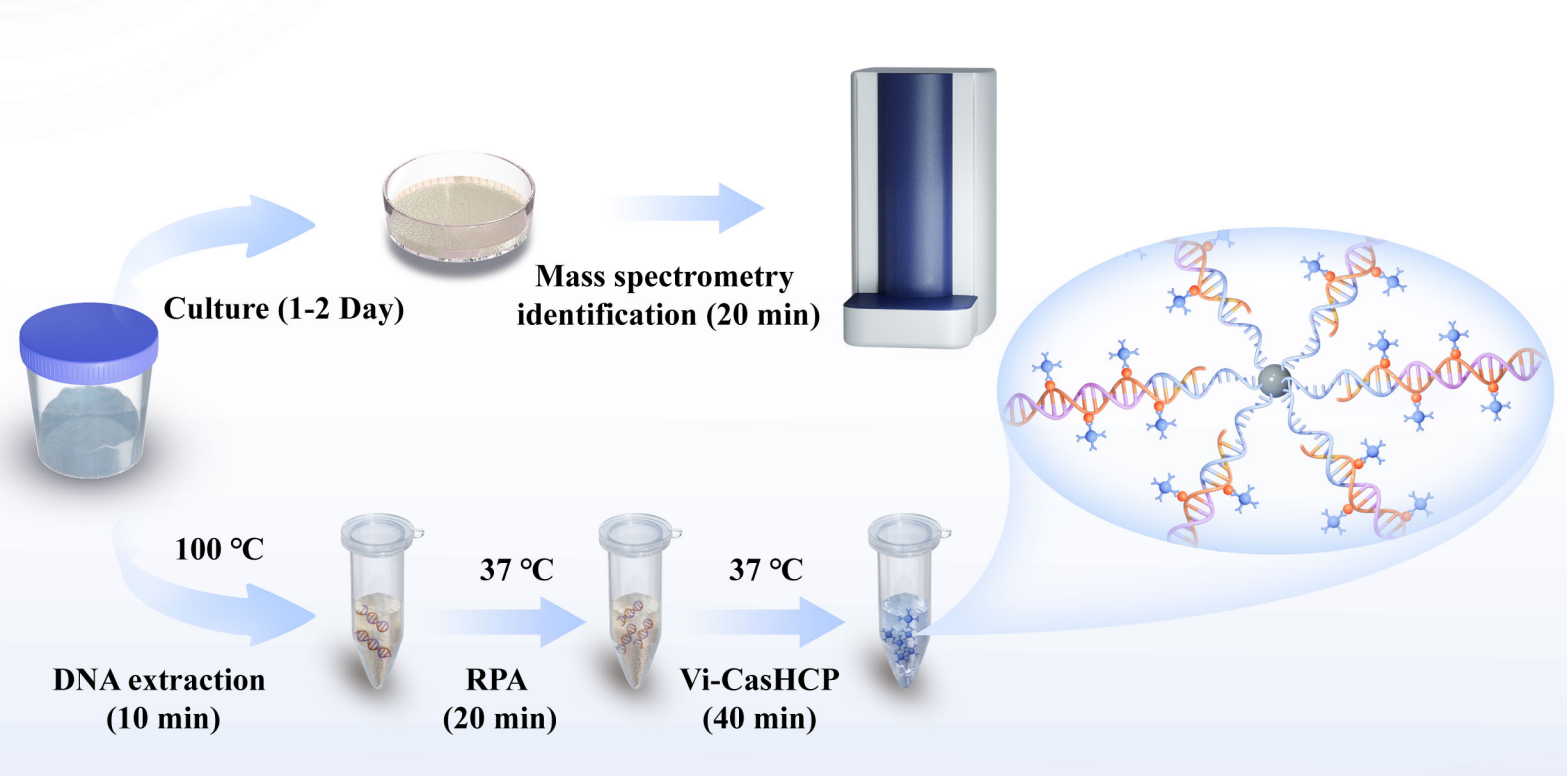
Schematic illustration of the Vi-CasHCP platform and culture/mass spectrometer-based *H. influenzae* test.

In addition, the Vi-CasHCP platform offers rapid detection and does not rely on large-scale instruments. In contrast, conventional culture-based identification of H. influenzae typically requires 1–2 days and often depends on expensive instruments, such as mass spectrometry, for species confirmation. The Vi-CasHCP detection process can be completed within 70 minutes, and the results can be interpreted visually (**Figure 5**).

### 3.5 Application in clinical sample testing

**Figure 6:**
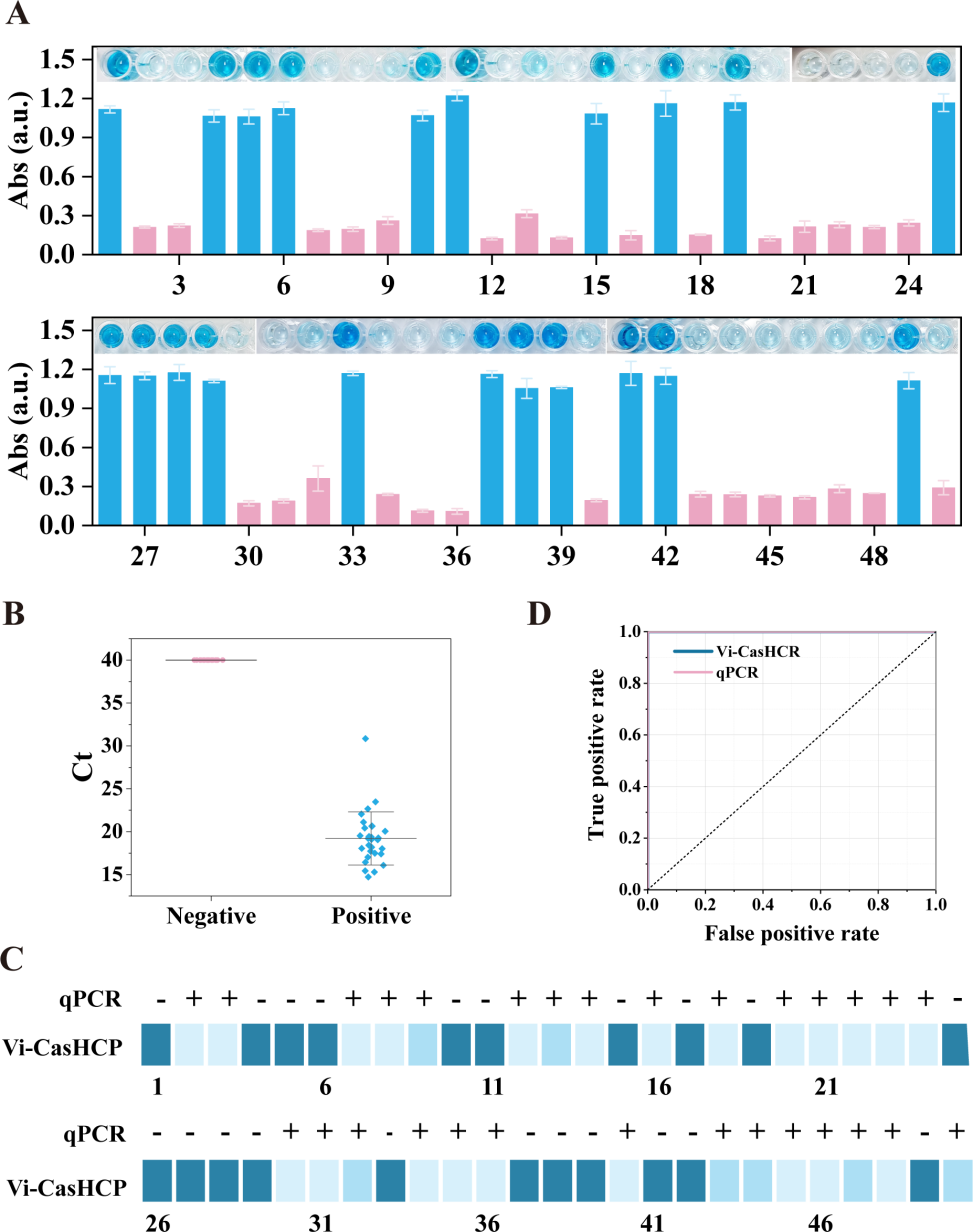
Application in clinical sample testing. **(A)** Detection results of 50 clinical samples. Top: photographs of colorimetric results; bottom: absorbance at 650 nm of Vi-CasHCP responses to 50 clinical samples. **(B)** Scatter plot analysis of the Ct value of qPCR for all 50 clinical samples. **(C)** Heatmap analysis of the Vi-CasHCP platform and qPCR method for 50 clinical samples. **(D)** ROC curve analysis of Vi-CasHCP for detecting *H. influenzae*, using qPCR as the reference standard.

To evaluate the clinical application potential of the Vi-CasHCP detection platform, we assessed its performance using clinical respiratory specimens. As shown in **Figure 6A**, Vi-CasHCP detected 29 positive and 21 negative cases, which can be easily identified with the naked eye. qPCR was used as the reference standard to confirm the accuracy and reliability of the Vi-CasHCP platform. We tested 50 clinically collected respiratory specimens using qPCR, with detailed amplification curves for each sample shown in **Supplementary Figure 8**. Negative samples cluster around Ct 40, indicating absence of target DNA, while positive samples range from approximately 15 to 30, showing clear separation (**Figure 6B**). A heatmap comparison of the Vi-CasHCP and qPCR detection results showed a high degree of consistency (**Figure 6C**). ROC curve analysis was performed for Vi-CasHCP using qPCR as the reference standard. Vi-CasHCP achieved an AUC of 1.00 (**Figure 6D**), indicating excellent diagnostic performance. Based on 50 clinical samples, including 29 true-positive and 21 true-negative samples, the sensitivity, specificity, positive predictive value (PPV), and negative predictive value (NPV) of Vi-CasHCP were all 100%. The 95% confidence intervals were 88%–100% for sensitivity and PPV, and 84%–100% for specificity and NPV. These results show that Vi-CasHCP not only offers accuracy comparable to traditional detection methods but also provides significant advantages in detection time and ease of use, making it a rapid, sensitive, and highly specific detection tool. Furthermore, Vi-CasHCP does not require complex instruments and can be performed using only simple and portable equipment such as a metal bath, pipettes, and a magnetic rack (**Supplementary Figure 9**).

To evaluate the reliability of the Vi-CasHCP visualization detection method, we developed a detailed reader guideline (in the supplementary materials) to clarify the standards and procedures for sample interpretation. Five independent observers were then invited to interpret all samples independently. During the interpretation process, the observers were blinded to the true status of the samples and determined each sample as negative or positive based on the colorimetric chart. The results showed complete agreement among all five observers across all 50 samples, indicating that the method is highly reliable (**Supplementary Table 7**). Therefore, Vi-CasHCP has significant potential for widespread clinical application, particularly for early screening of high-risk groups such as children and rapid testing in resource-limited clinical settings.

## Discussion

*H. influenzae* is primarily transmitted through close contact with individuals or respiratory secretions (Short et al., 2021; Koenen et al., 2024). It is a common pathogen that causes community-acquired respiratory infections, especially among children under five, who have a high infection rate (van de Beek et al., 2016). *H. influenzae* has stringent culture requirements, growing only on Chocolate Agar supplemented with growth factors in a carbon dioxide-rich environment. This fastidious nature often results in a low clinical isolation rate (Kaur and Pichichero, 2014). Serological and nucleic acid tests are also used to detect *H. influenzae*. However, neither fully meets the comprehensive clinical needs for speed, ease of use, and accuracy (Sohrabi et al., 2023).

We selected the *omp6* gene, which encodes the highly conserved *omp6* lipoprotein of *H. influenzae*, as the target for detection. A rapid visual colorimetric detection method, Vi-CasHCP, was developed by integrating RPA, the CRISPR/Cas12a system, HCR, and an HRP-catalyzed TMB-H_2_O_2_ colorimetric system. First, four pairs of RPA primers targeting the *omp6* gene sequence were synthesized and the primer with the best amplification efficiency was selected using agarose gel electrophoresis. Using the RPA amplicon as the target, three crRNAs were designed. The CRISPR/Cas12a fluorescent reporter system was then used to screen crRNA2, which exhibited the highest cleavage activity. HCR hairpin structures were designed using NUPACK software, and their assembly feasibility was confirmed via polyacrylamide gel electrophoresis. FAM-labeled DNA was used to confirm the successful functionalization of the carboxylated magnetic bead surface. Finally, the overall feasibility of the detection strategy was validated using both absorbance measurement and visual observation.

To enhance the detection performance of Vi-CasHCP, we systematically optimized key reaction conditions. We established the following optimal parameters: a Cas:crRNA molar ratio of 200 nM, a magnetic bead cutting time of 30 minutes, an HRP concentration of 0.4 μg, an H1/H2 concentration of 600 nM, an HCR reaction time of 7 minutes, and a TMB color development time of 3 minutes. Under these optimal conditions, we evaluated Vi-CasHCP’s detection performance. The results demonstrated that Vi-CasHCP exhibited good sensitivity, with a detection limit of 11.8 CFU/mL for *H. influenzae*. In specificity evaluation, Vi-CasHCP clearly distinguished *H. influenzae* from *P. aeruginosa*, *S. pneumoniae*, *E. coli*, *S. pyogenes*, *K. pneumoniae*, *S. aureus*, and *M. catarrhalis*, as well as their mixed strains. To further assess its applicability in clinical samples, we collected 10 clinically isolated *H. influenzae* strains, 10 common respiratory pathogens, and 50 clinical respiratory specimens. Parallel detection was performed using both Vi-CasHCP and qPCR methods. The results indicated that Vi-CasHCP not only demonstrated detection accuracy comparable to qPCR but also offered significant advantages in detection speed and reduced instrument dependence, highlighting its potential for clinical application.

Vi-CasHCP, a novel detection method, offers significant advantages in detecting *H. influenzae*. First, by integrating magnetic separation technology, this method facilitates convenient purification and component separation, effectively reducing matrix interference and enhancing operational stability. Secondly, due to the quadruple cascade amplification comprising RPA amplification, Cas12a enzyme digestion, HCR signal amplification, and HRP-catalyzed color development, Vi-CasHCP achieves ultra-high sensitivity, detecting as low as 11.8 CFU/mL. In addition, the assay requires only simple equipment, such as a metal bath and a magnetic rack, thereby reducing instrument dependence and making it more suitable for resource-limited settings.

Despite the superior performance of Vi-CasHCP, the current workflow remains a multi-step procedure that relies on manual handling, which may introduce operational errors. Repeatedly opening reaction tubes may also increase the risk of cross-contamination and aerosol generation. Therefore, careful handling and strict procedural controls are necessary to minimize these risks. Additionally, the study is limited by the relatively small number of clinical samples analyzed, which may not fully reflect strain variability or the method’s potential clinical applicability. In future work, to enhance the method’s integration and automation, we will explore combining it with microfluidic chip technology (Li et al., 2026) (Shen et al., 2023). By physically isolating reaction modules, such as nucleic acid extraction, target recognition, and signal output, in different micro-regions of the chip, the system is expected to achieve “one-card” operation, allowing the entire detection process to be completed with a single sample addition (Xie et al., 2025). This will greatly simplify operation steps, improve detection throughput, and enhance result reproducibility. Future studies should also include larger sample sizes and more diverse specimen types to further validate the robustness and clinical utility of this method.

In summary, Vi-CasHCP offers significant advantages for detecting *H. influenzae*, including ultra-high sensitivity, no need for complex instrumentation, excellent accuracy, and ease of use. Leveraging the editability of crRNA, this method can be extended to detect other pathogenic microorganisms. The innovative Vi-CasHCP technology has significant potential in fields such as infectious disease diagnosis, public health, environmental monitoring, and food safety.

## Data Availability

The original contributions presented in the study are included in the article/**Supplementary Material**. Further inquiries can be directed to the corresponding author.
