# Correction: Hybridization chain reaction-assisted CRISPR/Cas12a strategy for rapid and visual detection of *Haemophilus influenzae*

**DOI:** 10.3389/fcimb.2026.1936162

**Published:** 2026-08-04

**Authors:** Chunfang Ma, Jiayi Zhang, Yayun Jiang, Xiangxiang Li

**Affiliations:** 1 Department of Clinical Laboratory, Suzhou Ninth People’s Hospital, Suzhou Ninth Hospital Affiliated to Soochow University, Suzhou, China; 2 Department of Obstetrics, Suzhou Ninth People’s Hospital, Suzhou Ninth Hospital Affiliated to Soochow University, Suzhou, China; 3 Department of Clinical Laboratory, Deyang People′s Hospital, Deyang, China

**Keywords:** CRISPR/Cas12a, *Haemophilus influenzae*, hybridization chain reaction, rapid, visual detection

There was an omission in the caption of **Figure 1** as published. The original caption did not include a detailed explanation of the components and workflow illustrated in the schematic diagram, which may affect readers’ understanding of the detection principle. The corrected caption of **Figure 1** appears below.

**Figure 1:**
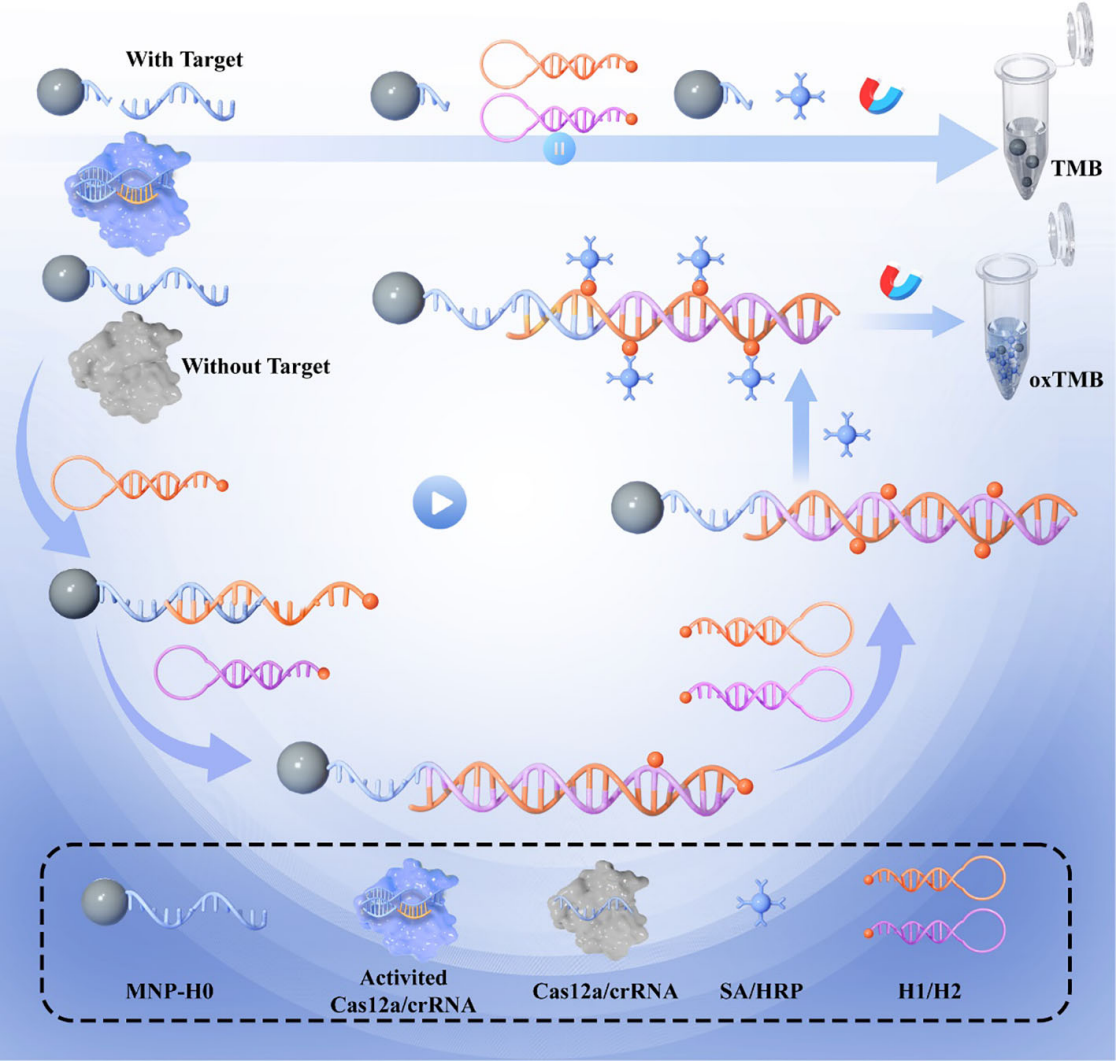
Schematic illustration of the Vi-CasHCP platform.

The original version of this article has been updated.

