# Supplementary material for: Hybridization chain reaction-assisted CRISPR/Cas12a strategy for rapid and visual detection of Haemophilus influenzae
Source: Front Cell Infect Microbiol. 2026 Jul 7;16:1844708. doi: 10.3389/fcimb.2026.1844708 (PMC13384864; doi:10.3389/fcimb.2026.1844708)
Supplement: Supplementary file 1 [file DataSheet1.pdf]

Supplementary material

Hybridization Chain Reaction-Assisted CRISPR/Cas12a Strategy for Rapid and Visual Detection of *Haemophilus influenzae*

Chunfang Ma[1†], Jiayi Zhang[2†], Yayun Jiang[3*], and Xiangxiang Li[1*]

[1] Department of Clinical Laboratory, Suzhou Ninth People's Hospital, Suzhou Ninth Hospital Affiliated to Soochow University, Suzhou, Jiangsu, China

[2] Department of Obstetrics, Suzhou Ninth People's Hospital, Suzhou Ninth Hospital Affiliated to Soochow University, Suzhou, Jiangsu, China

[3] Department of Clinical Laboratory, Deyang People′s Hospital, Deyang, Sichuan, China.

[*]Corresponding authors: dyjiangyayun@4163.com (Yayun Jiang) and lxx1991@suda.edu.cn (Xiangxiang Li),

[†]Chunfang Ma and Jiayi Zhang contributed equally to this work.

Table S1. The sequences of crRNA and primer are used in this work.

Names	Sequences (5'-3')
RPA-F1(*P6*)	TAGCTGCTTGTAGTTCCTCTAAC
RPA-R1(*P6*)	CCTAATGCGATGTTGTATTCTGG
RPA-F2(*P6*)	GGATACTCTGTTGCTGATCTTCA
RPA-R2(*P6*)	CCTAATGCGATGTTGTATTCTGG
RPA-F3(*P6*)	TAGCTGCTTGTAGTTCCTCTAAC
RPA-R3(*P6*)	CACCGTAAGATACTGTGCCTAAT
RPA-F4(*P6*)	ACTTTTGGCGGATACTCTGT
RPA-R4(*P6*)	TGTGCCTAATTTACCAGCAT
crRNA1	UAAUUUCUACUAAGUGUAGAUAAUGCAACGCCAGCUGCUAA
crRNA2	UAAUUUCUACUAAGUGUAGAUGCGGAUACUCUGUUGCUGAU
crRNA3	UAAUUUCUACUAAGUGUAGAUAUAAAUACGACAUCACCGGU

Table S2. The sequences of oligonucleotides are used in this work.

Names	Sequences (5’-3’)
H0	TTTTTTTTTTTTAGAAGAAGGTGTTTAAGT
H1	TTTTTTGTTTAAGTTGGAGAATTGACTTAAACACCTTCTTCT
H2	CAATTCTCCAACTTAAACAGAAGAAGGTGTTTAAGTTTTTTT
NH_2-H0	NH_2-TTTTTTTTTTTTAGAAGAAGGTGTTTAAGT
Biotin-H1	Biotin-TTTTTTGTTTAAGTTGGAGAATTGACTTAAACACCTTCTTCT
H2-Biotin	CAATTCTCCAACTTAAACAGAAGAAGGTGTTTAAGTTTTTTT-Biotin

Table S3 Information on clinically isolated strains used in this study.

Sample number	Specimen type	Clinical test results
S1	Sputum	*P. aeruginosa*
S2	Sputum	*S. pneumoniae*
S3	Sputum	*K. pneumoniae*
S4	Sputum	*M. catarrhalis*
S5	Sputum	*M. catarrhalis*
S6	Throat Swabs	*E. coli*
S7	Throat Swabs	*S. aureus*
S8	Throat Swabs	*E. coli*
S9	Bronchoalveolar lavage	*S. pneumoniae*
S10	Bronchoalveolar lavage	*K. pneumoniae*
S11	Secreta	*H. influenzae*
S12	Throat Swabs	*H. influenzae*
S13	Bronchoalveolar lavage	*H. influenzae*
S14	Throat Swabs	*H. influenzae*
S15	Throat Swabs	*H. influenzae*
S16	Sputum	*H. influenzae*
S17	Sputum	*H. influenzae*
S18	Vaginal swab	*H. influenzae*
S19	Bronchoalveolar lavage	*H. influenzae*
S20	Sputum	*H. influenzae*

Table S4 Clinical information for the 50 respiratory samples used in this study.

Sample number	Specimen type	Age	Clinical diagnosis	Culture + MALDI-TOF
1	Sputum	28 days	Pneumonia	*S. aureus*
2	Sputum	4 months	Bronchopneumonia	*H. influenzae*
3	Sputum	9 months	Pneumonia	*H. influenzae*
4	Sputum	3 years	Pneumonia	*M. catarrhalis*
5	Sputum	5 years	Pneumonia	*M. catarrhalis*
6	Sputum	3 years	Pneumonia	*M. catarrhalis*
7	Sputum	3 years	Bronchopneumonia	*H. influenzae*
8	Sputum	4 years	Bronchopneumonia	*H. influenzae*
9	Sputum	3 years	Pneumonia	*H. influenzae*
10	Sputum	3 years	Pneumonia	*S. pneumoniae*
11	Sputum	3 years	Pneumonia	*M. catarrhalis*
12	Sputum	3 years	Pneumonia	*H. influenzae*
13	Sputum	2 years	Fever of unknown origin	*H. influenzae*
14	Sputum	3 years	Pneumonia	*H. influenzae*
15	Sputum	5 years	Pertussis pneumonia	*M. catarrhalis*
16	Sputum	4 years	Pneumonia	*H. influenzae*
17	Sputum	2 years	Bronchopneumonia	*S. pneumoniae*
18	Sputum	5 years	Bronchopneumonia	*H. influenzae*
19	Sputum	4 months	Pneumonia	*S. pneumoniae*
20	Sputum	1 month	Pneumonia	*H. influenzae*
21	Sputum	11 months	Pneumonia	*H. influenzae*
22	Sputum	4 years	Bronchopneumonia	*H. influenzae*
23	Sputum	3 years	Bronchopneumonia	*H. influenzae*
24	Sputum	3 years	Pneumonia	*H. influenzae*
25	Sputum	2 years	Pneumonia	*S. pneumoniae*
26	Sputum	3 years	Bronchopneumonia	*S. pneumoniae*
27	Sputum	3 years	Pneumonia	*M. catarrhalis*
28	Sputum	3 years	Pneumonia	*S. pneumoniae*
29	Sputum	3 years	Pneumonia	*M. catarrhalis*
30	Sputum	4 years	Pneumonia	*H. influenzae*
31	Sputum	4 years	Bronchopneumonia	*H. influenzae*

32	Sputum	2 months	Bronchopneumonia	*H. influenzae*
33	Sputum	13 days	Bronchopneumonia	*E. coli*
34	Sputum	4 years	Pneumonia	*H. influenzae*
35	Sputum	4 years	Bronchopneumonia	*H. influenzae*
36	Sputum	1 year	Bronchopneumonia	*H. influenzae*
37	Sputum	5 years	Pneumonia	*S. pneumoniae*
38	Sputum	3 years	Bronchopneumonia	*S. pneumoniae*
39	Sputum	7 years	Pneumonia	*S. aureus*
40	Sputum	3 years	Pneumonia	*H. influenzae*
41	Sputum	2 years	Bronchopneumonia	*M. catarrhalis*
42	Sputum	3 years	Pneumonia	*S. pneumoniae*
43	Sputum	4 years	Pneumonia	*H. influenzae*
44	Sputum	2 years	Pneumonia	*H. influenzae*
45	Sputum	1 year	Bronchopneumonia	*H. influenzae*
46	Sputum	10 years	Bronchopneumonia	*H. influenzae*
47	Sputum	8 years	Pneumonia	*H. influenzae*
48	Sputum	13 years	Pneumonia	*H. influenzae*
49	Sputum	11 years	Fever of unknown origin	*S. aureus*
50	Sputum	13 years	Pneumonia	*H. influenzae*

Table S5 A650 values from repeated low-concentration testing of *H. influenzae*

Replicate No.	12 CFU/ml	9 CFU/ml	6 CFU/ml	3 CFU/ml
1	0.5818	0.6531	0.7511	**1.1582**
2	0.6256	0.6902	0.779	0.7508
3	0.5906	0.6667	**1.047**	**1.1095**
4	0.6581	**1.2071**	**1.1572**	**1.196**
5	0.6368	0.7055	0.7546	**1.1301**
6	0.6401	0.7005	0.7231	**1.1154**
7	0.638	0.6971	0.7373	**1.1332**
8	0.6088	0.6691	**1.1035**	0.6717
9	0.6053	0.6707	0.6955	**1.1005**
10	0.6142	0.6672	0.6986	**1.0845**
11	0.6742	0.7613	**1.1124**	**1.247**
12	0.6204	0.6863	0.748	**1.1363**
13	0.6159	0.6925	**1.2041**	**1.2234**
14	0.6406	0.691	0.7172	**1.1142**
15	0.6041	0.6026	0.6428	**1.0644**
16	0.6085	**1.2513**	0.9676	0.5521
17	0.6038	0.6556	0.6926	**1.1147**
18	0.6049	0.6694	0.6612	**1.0671**
19	0.6047	0.6744	**1.0301**	**1.1435**
20	0.6285	**1.1136**	**1.0879**	0.6202

Table S6 LOD evaluation of Vi-CasHCP based on a ≥95% detection probability criterion.

**Concentration (CFU/mL)**	**No. positive/ No. tested (*H. influenzae*)**	**Positive Ratio**
12	20/20	1.00
9	17/20	0.85
6	13/20	0.65
3	4/20	0.20
LOD	-	11.8 CFU/mL (95% CI: 9.8 – 14.3)

Table S7: Inter-Observer Consistency of Vi-CasHCP Detection Results of 50 clinical samples

Sample ID	Observer 1	Observer 2	Observer 3	Observer 4	Observer 5	Agreement
**1**	-	-	-	-	-	YES
**2**	+	+	+	+	+	YES
**3**	+	+	+	+	+	YES
**4**	-	-	-	-	-	YES
**5**	-	-	-	-	-	YES
**6**	-	-	-	-	-	YES
**7**	+	+	+	+	+	YES
**8**	+	+	+	+	+	YES
**9**	+	+	+	+	+	YES
**10**	-	-	-	-	-	YES
**11**	-	-	-	-	-	YES
**12**	+	+	+	+	+	YES
**13**	+	+	+	+	+	YES
**14**	+	+	+	+	+	YES
**15**	-	-	-	-	-	YES
**16**	+	+	+	+	+	YES
**17**	-	-	-	-	-	YES
**18**	+	+	+	+	+	YES
**19**	-	-	-	-	-	YES
**20**	+	+	+	+	+	YES
**21**	+	+	+	+	+	YES
**22**	+	+	+	+	+	YES
**23**	+	+	+	+	+	YES
**24**	+	+	+	+	+	YES
**25**	-	-	-	-	-	YES
**26**	-	-	-	-	-	YES
**27**	-	-	-	-	-	YES
**28**	-	-	-	-	-	YES
**29**	-	-	-	-	-	YES
**30**	+	+	+	+	+	YES

**31**	+	+	+	+	+	YES
**32**	+	+	+	+	+	YES
**33**	-	-	-	-	-	YES
**34**	+	+	+	+	+	YES
**35**	+	+	+	+	+	YES
**36**	+	+	+	+	+	YES
**37**	-	-	-	-	-	YES
**38**	-	-	-	-	-	YES
**39**	-	-	-	-	-	YES
**40**	+	+	+	+	+	YES
**41**	-	-	-	-	-	YES
**42**	-	-	-	-	-	YES
**43**	+	+	+	+	+	YES
**44**	+	+	+	+	+	YES
**45**	+	+	+	+	+	YES
**46**	+	+	+	+	+	YES
**47**	+	+	+	+	+	YES
**48**	+	+	+	+	+	YES
**49**	-	-	-	-	-	YES
**50**	+	+	+	+	+	YES

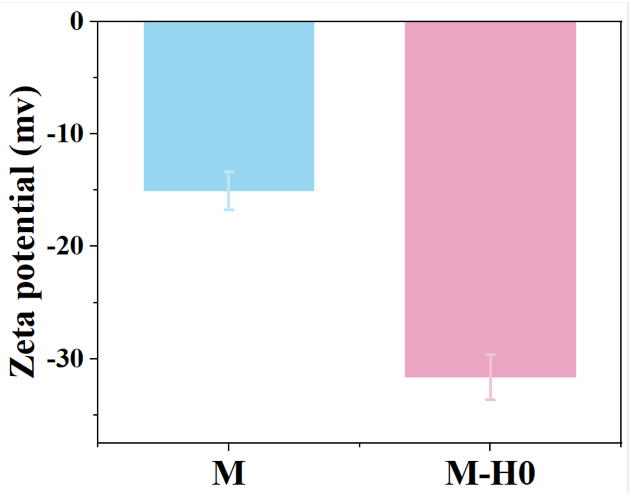

Figure S1. Zeta potential measurements of magnetic beads before (M) and after (M-H0) DNA conjugation.

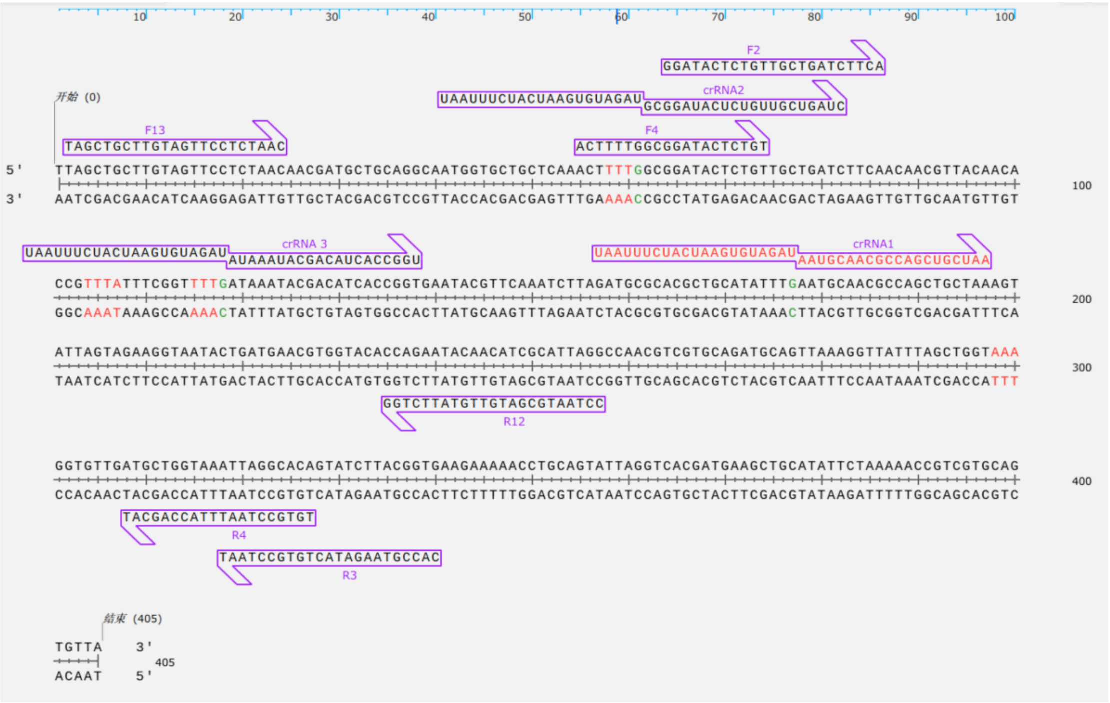

Figure S2. Primer sequences, crRNA sequences, and binding sites used for RPA primer and CRISPR/Cas12a system screening.

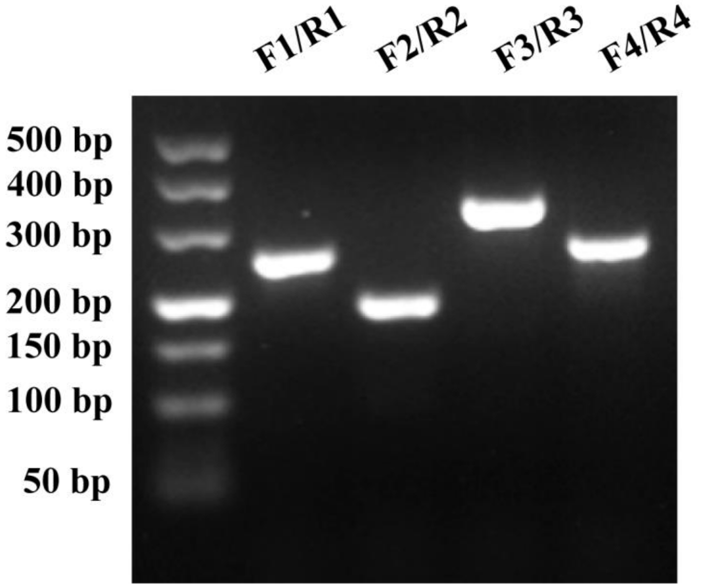

Figure S3. Screening of RPA primer pairs targeting the *omp6* gene by 3% agarose gel electrophoresis.

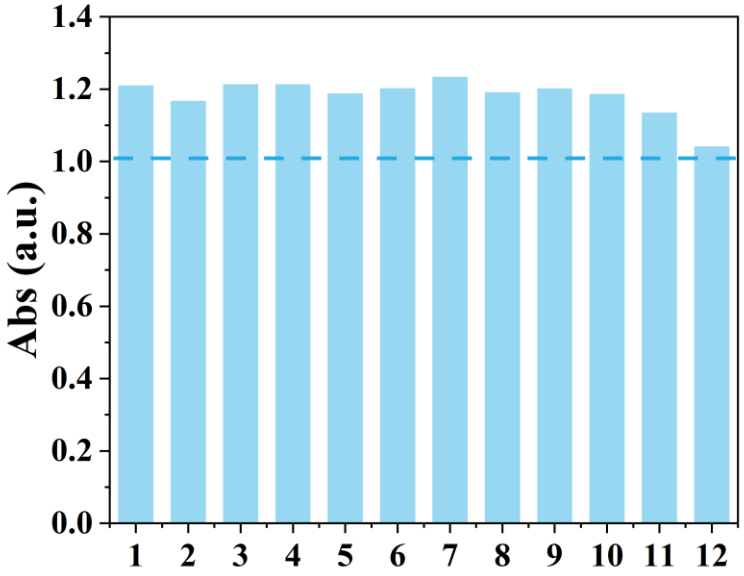

Figure S4. Calculate the cut-off value for the absorbance signal of the Vi-CasHCP. X - 3SD calculates the cutoff value, where X (1.18) is the average signal value from 12 replicates without the target gene, and SD (0.05) is the standard deviation.

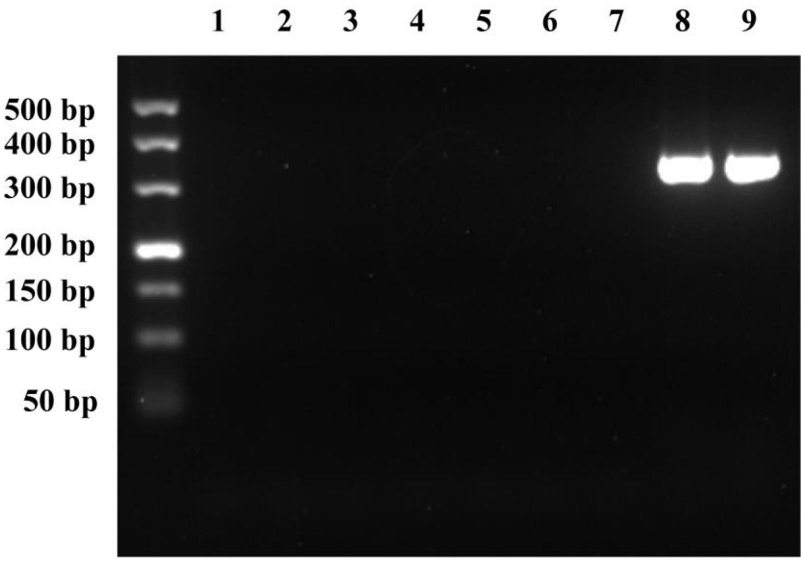

Figure S5. 3% agarose gel electrophoresis was used to verify the specificity of the Vi-CasHCP platform detection for different standard strains.: line 1−9: *P. aeruginosa* (ATCC27853), *S. pneumoniae* (ATCC49619), *E. coli* (ATCC 25922), *S. pyogenes* (ATCC 19615), *K. pneumoniae* （ATCC700603）, and *S. aureus* (ATCC29213). M. catarrhalis (ATCC 25240), H. influenzae (ATCC 9007), and all strains mixed.

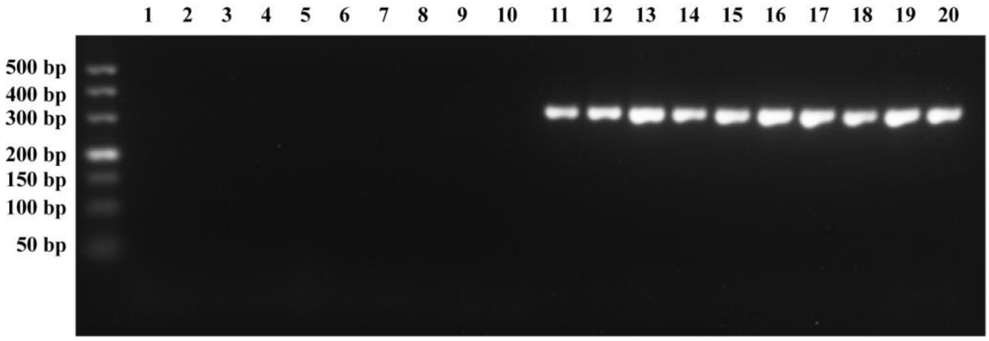

Figure S6. (A) 3% agarose gel electrophoresis analysis of *omp6* gene amplification in different clinical bacterial isolates. line 1-10: *P. aeruginosa, S. pneumoniae, K. pneumoniae, M. catarrhalis, M. catarrhalis, E. coli, S. aureus, E. coli, S. pneumoniae, K. pneumoniae*. line 11−20:10 strains of *H. influenzae.*

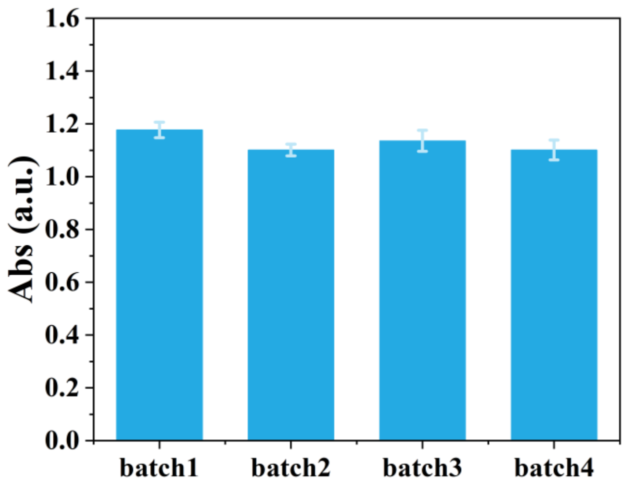

Figure S7. Batch-to-batch reproducibility of M-H0. Absorbance values at 650 nm of four independently prepared M-H0 batches. Error bars represent the standard deviation from three independent measurements.

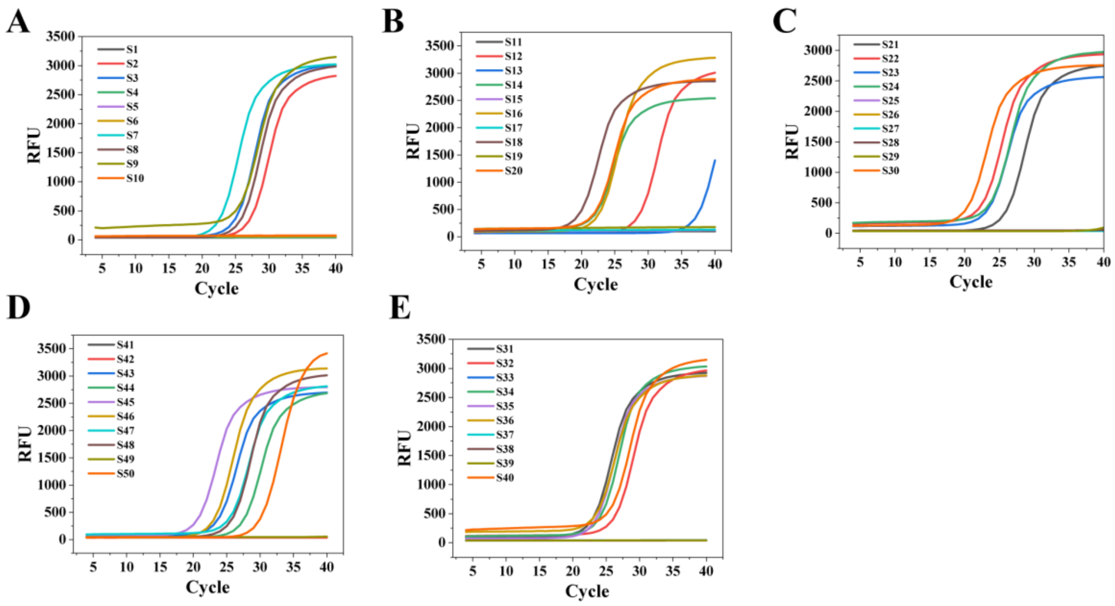

Figure S8. qPCR for the detection of clinical respiratory specimens.

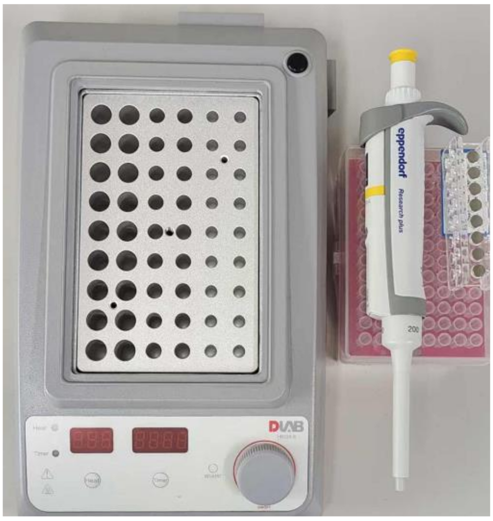

Figure S9. The instruments required for the Vi-CasHCP platform.

Supplementary Method S1 Vi-CasHCP Visual Detection Guideline

Purpose: This guideline is intended to assist laboratory and clinical personnel in interpreting Vi-CasHCP assay results using the provided colorimetric reference card.

Steps:

- Observation: Place the completed reaction well (or reaction tube) on a white background and lay it flat alongside the colorimetric card.
- Comparison: Observe the colorimetric card from left to right and identify the reference well whose color is closest to that of the sample.
- Determination: Determine the test result according to the following interpretation criteria.

Colorimetric Reference Card:

- Negative (-): Dark blue, high absorbance
- Positive (+): Gradually lighter blue for higher concentrations
- Weakly positive (±): Repeat testing is recommended, and absorbance at 650 nm should be measured to further confirm the reliability of the result.

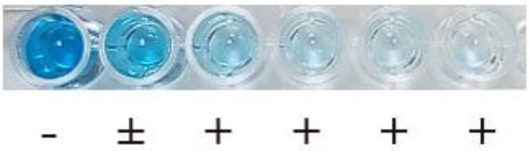

Colorimetric reference card

Operational Notes:

- The interpretation should be performed within the specified time after completion of the reaction to avoid color changes caused by prolonged standing.
- The colorimetric card is only applicable to this assay kit and should not be replaced with any other color card.
- If abnormal colors appear (e.g., turbidity, precipitation, or bubbles), the test should be repeated.
- It is recommended that the results be interpreted by trained personnel and properly documented.
